# Words, images and gender: Lessons from a survey on the public perception of synthetic biology and related disciplines

**DOI:** 10.15252/embr.201948401

**Published:** 2019-06-26

**Authors:** Manuel Porcar, Adriel Latorre‐Pérez, Esther Molina‐Menor, Martí Domínguez

**Affiliations:** ^1^ Institute for Integrative Systems Biology (I2SysBio) Universitat de València‐CSIC Valencia Spain; ^2^ Darwin Bioprospecting Excellence SL Valencia Spain; ^3^ Language Theory and Communication Sciences Department (UV) Universitat de València Valencia Spain

**Keywords:** Synthetic Biology & Biotechnology, S&S: Economics & Business, S&S: Politics, Policy & Law

## Abstract

A large survey of visitors at a science museum about the perception of biotechnology shows that names matter and that gender has an influence on people's attitude towards new technologies.

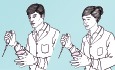

The fast development of new research fields, such as genetic engineering or synthetic biology, is often met with public concerns or even resistance, the fate of genetically modified crops being a prime example. There are many factors at play that determine how laypeople perceive new technologies and a better understanding of these can help to inform debate. Foremost, however, it is necessary to obtain reliable information on public opinion of emerging technologies that have the potential to affect their lives. To this end, we conducted a survey to gauge public opinion on genetic engineering and biotechnology as part of a special exhibition at the CosmoCaixa Museum in Barcelona, Spain. The large sample size of 38,113 respondents allowed us to assess the effect of age, gender or education on the perception of three related terms: “biotechnology”, “genetic engineering” and “synthetic biology”. In addition, by randomly associating these terms with the image of either a male or a female scientist, we looked at the effect of gender on people's perception of these technologies. In short, two conclusions can be reached: the terms “biotechnology” and “genetic engineering” were preferred to “synthetic biology”. Second, terms associated with an image of a female scientist were better rated compared to the same terms associated with a male researcher. These results show an interesting gender dimension of public perception of new technologies.

## Public perception of biotechnology

Synthetic biology, genetic engineering and biotechnology are interrelated terms with blurred boundaries. Biotechnology uses living organisms, cells or cellular components to synthesize products for agriculture, medicine, industry and research and has been used for centuries, albeit unconsciously. Genetic engineering is one of the subdisciplines of biotechnology: it involves the manipulation of an organism's DNA sequence by addition, deletion or modification in order to expand the product range of biotechnology. While both generally are based on using organisms, genes or metabolic pathways from nature, synthetic biology aims to design novel artificial systems. Synthetic biology can thus be seen as both an extension of genetic engineering, as well as a new view on biotechnology by using engineering principles such as standardization, modularity or orthogonality 1.

There are many factors at play that determine how laypeople perceive new technologies and a better understanding of these can help to inform debate.

In the public eye, however, biotechnology, genetic engineering and synthetic biology are often reduced to genetically modified organisms (GMOs). This, combined with a critical perception of GMOs, has fuelled a generally negative attitude of biotechnology. The last Eurobarometer survey (2010) on GM food showed that only 5% of Europeans completely support it, 18% “tend to agree”, but as much as 61% totally disagree, that is, are against GM food. Moreover, 83% of Europeans had not heard about synthetic biology before. The main concerns were the possible risks rather than potential benefits from these technologies (http://ec.europa.eu/commfrontoffice/publicopinion/index.cfm/Survey/index#p=1&instruments=SPECIAL&search=341). Indeed, genetic engineering is perceived with a higher degree of concern compared to other scientific fields 2.

… Generation T (2011–present), also known as Generation Alpha, is growing up with an iPad or a smartphone in their hand in front of a screen.

In relation to perceptions of gender, a number of recent studies have shown biases of how men and women are evaluated and perceived at work 3, 4. A randomized double‐blind study of professors in biology, chemistry and physics showed that identical academic profiles were more positively evaluated when they belonged to a male student than a female student. The result of such biases is that women in academia have to work harder than their male peers to obtain the same recognition 5 and that males are often seen as more capable than women 6. Just to highlight one common example of gender stereotyping, when using neutral or non‐gender‐specific language, people tend to assume that a specialist in question is a man 7.

## The exhibition and the survey

The survey was carried out in the CosmoCaixa museum, a flagship science museum in Barcelona that is sponsored by La Caixa Banking Foundation. Entry is free for students younger than 18 and for CaixaBank customers; visitors can explore a variety of permanent and temporary exhibitions, including “Top Ciència” (Top Science), which features emerging technologies and research areas. It is a small part of the museum with a few stands, mainly screen‐based. From November 2016 to April 2018, Top Ciència hosted an exhibition on biotechnology, coordinated by one of the authors (MP) (https://blog.caixaciencia.com/ca/-/biologia-sintetica-que-vienen-las-biomaquinas). It included videos in which researchers explained synthetic biology; a display of the *E. chromi* suitcase designed by Alexandra Daisy Ginsberg featuring plastic replicas of human stool (https://www.daisyginsberg.com/work/echromi-living-colour-from-bacteria); and a set of interactive screens to further explain synthetic biology. One of these screens was used for our survey.

Interestingly, women tended to evaluate female images more positively than male images …

**Figure 1:**
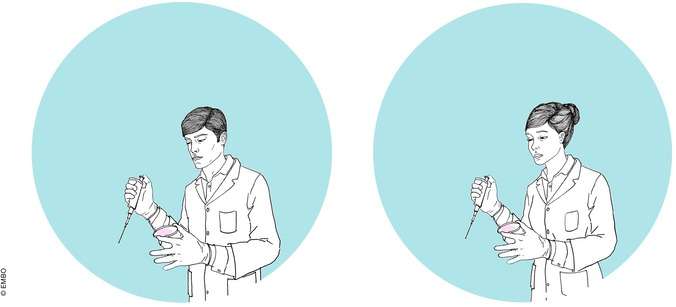
**Images of a male scientist (left) and a female scientist (right) shown to the respondents**.

The survey was available in Spanish, Catalan and English, and asked participants about their age, gender, education followed by the crucial question “How would you rate the following scientific field?” from completely unfavourable (0) to totally favourable (10). The term displayed was either “synthetic biology”, “genetic engineering” or “biotechnology”, and it was always associated with an image of either a female or a male scientist (Fig 1). The three terms and two images yielded a total of six combinations, only one of which was randomly displayed and rated by the respondent. Finally, the screen provided respondents with feedback showing a summary of the results.

We classified the answers based on the gender of the respondent, age and level of education—from primary school to university studies. The age groups were defined according to the generation they belonged to. Although there is no consensus on the exact limit between generations, based on the literature 8 and the data collected, we divided the respondents into five age groups. The silent Generation, born between 1920 and 1945, had experienced wars and international conflicts, such as World War II and, in the case of Spain, the Civil War from 1936 to 1939, and Franco's dictatorship. They were “silent” because of the risk that expressing one's opinion involved. The baby boomers, born between 1946 and 1964, are the largest group. Generation X, born between 1965 and 1980, experienced important social changes such as the Cold War. In Spain, they lived through the transition from dictatorship to democracy. Generation Y or millennials were born between 1981 and 1996. They are strongly influenced by the huge technological developments starting in the 1980s and grew up with computers and ever more sophisticated electronic devices. Generation Z or centennials (1997–2010) take for granted the Internet, social media and technologies in their daily life. They have been growing up in a globalized world, and their social skills have adapted to their use of digital devices. Finally, Generation T (2011–present), also known as Generation Alpha, is growing up with an iPad or a smartphone in their hand in front of a screen.

The higher the level of education, the higher the appreciation of synthetic biology, biotechnology or genetic engineering.

We obtained a total of 38,113 valid answers from 17,284 men and 20,829 women (1,632 from the Silent Generation; 1,265 from baby boomers; 4,455 from Generation X; 6,160 from Generation Y or millennials; 18,840 from Generation Z or centennials; and 5,761 from Generation T). Their age ranged from 1 to 110 years old—both extremes being dubious for obvious reasons, and the reason why Generation T answers were not taken into account for the analysis. A total of 11,979 respondents had a university education; 3,221 had professional training; 9,276 had secondary school studies; and 13,637 had finished primary school.

## Perceptions of biotechnology and gender

**Figure 2:**
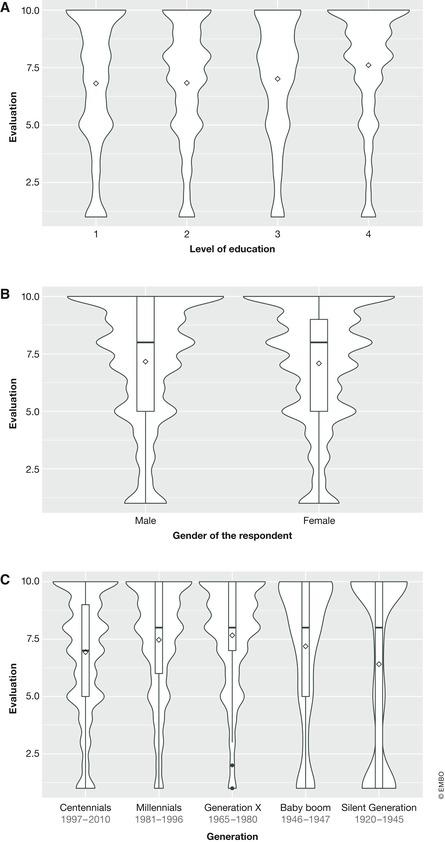
Perception of the disciplines based on level of education, gender and age (A) Violin plot showing the differences in evaluation based on the level of education. (B) Violin plot showing the differences in evaluation based on the gender of the respondent. (C) Violin plot showing the differences in evaluation based on the age of the respondent. The *Y* axis represents the score given in each category. The average value is represented by a diamond shape. The width/amplitude of the violin is proportional to how often that value has been selected by respondents.

Almost half of the responses (49.43%) came from the centennial generation born between 1997 and 2010. The highest average value was given by Generation X, followed by millennials and baby boomers. The lowest average values came from the Silent Generation (Fig 2C). The overall average value assigned to any of the three terms by men was 7.163, whereas the average value given by women was 7.086 (Fig 2B). The results also show that the higher the level of education, the higher the approval (Fig 2A). People holding a university degree gave an average evaluation of 7.606, whereas those with primary or secondary studies assigned 6.810 and 6.8232, respectively.

**Figure 3:**
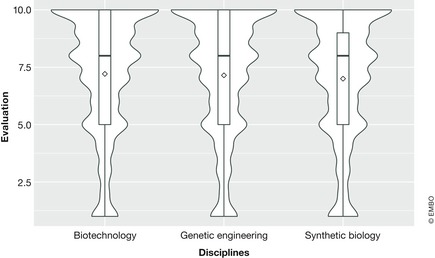
Perception of the disciplines in general The *Y* axis represents the evaluations. The average value is represented by a diamond shape. The width/amplitude of the violin is proportional to how often the value has been selected by the respondents.

There were significant differences in the average values for each discipline (Fig 3). Biotechnology was rated best with 7.210, whereas synthetic biology scored last (7.001) and genetic engineering scored 7.147. The results suggest a surprisingly similar rating of the three disciplines with a slight preference for biotechnology/genetic engineering over synthetic biology, which is a bit puzzling given that the exhibition featured synthetic biology.

As expected, there were also differences depending on the age of the respondents. The average values for each discipline were highest for millennials and Generation X, and the lowest for the Silent Generation. Synthetic biology was the lowest‐valued discipline in all age ranges, whereas biotechnology was better perceived by centennials and millennials. Although the distributions were similar, the average values tend to increase with the level of education.

**Figure 4:**
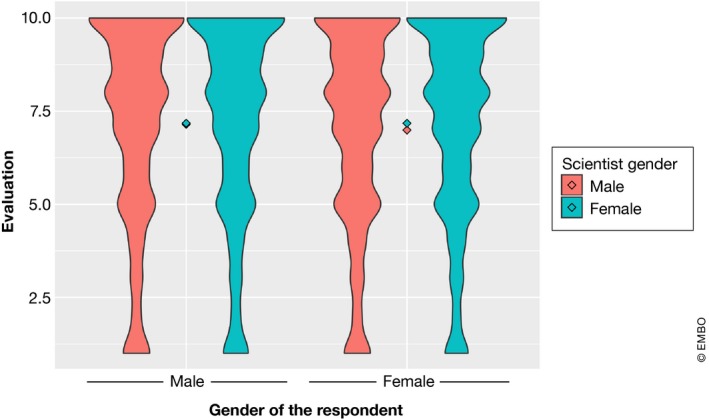
Gender‐depending perception Violin plot showing the differences in gender perception based on the gender of the respondent. The *Y* axis represents the evaluation of the man/woman scientist image. The average value is represented by a diamond shape. The width/amplitude of the violin is proportional to how often the value has been selected by the respondents.

Interestingly, women tended to evaluate female images more positively than male images, whereas men showed no significant difference in how they rated male versus female images. Moreover, women gave a lower average value to male images (6.993) than men (7.148; Fig 4). In the case of a female scientist being displayed, men rated it with 7.178 (an almost identical value given to male images), whereas women rated female researcher‐associated disciplines with 7.176.

## Conclusions

There are two main conclusions related to the terminology and to gender perceptions. The survey indicates that the sample population clearly prefers the terms “biotechnology” or “genetic engineering” over “synthetic biology”. This is unexpected, since the context in which the survey was carried out—a science museum with a temporary exhibition describing synthetic biology—was expected to induce a more positive view of synthetic biology. The results suggest that either the exhibition did not succeed in changing people's views of synthetic biology or that the public's negative views cannot easily be swayed. Interestingly, there was no significant difference in public attitudes between biotechnology and genetic engineering although the latter more easily invokes notions of GMOs.

Similarly interesting and somewhat unexpected is the finding that disciplines associated with a female researcher were better rated than disciplines associated with a male researcher, and that this trend is particularly strong for female respondents. Since the images were displayed randomly and since only one image was shown to each respondent, the differences in rating could be explained by an unconscious preference by women for female scientists, whereas men are strikingly indifferent to the gender displayed when it comes to rating a scientific discipline associated with an image. Alternatively, it might be explained by a bias in favour of female scientists at least among the women attending a scientific museum; in other words, the sample population might have been enriched for women who are interested in and motivated by science. Lastly, given that the vast majority of visitors and respondents were young—born before 1997—the bias of women in favour of female scientists could also be linked to a more positive identification with the depicted character after years of outreach efforts to promote women in science. Whatever, the reason(s), we think that this is a solid effect given the size and design of our survey, which would warrant more studies and analysis of gender‐based perceptions of scientists.

In addition, we found a difference in perceptions of both gender and discipline depending on education. The higher the level of education, the higher the appreciation of synthetic biology, biotechnology or genetic engineering. This observation matches with a recent paper in *Nature Human Behavior* 9 about GMO perception, which concludes that the less people know about GMOs and the science behind it, the stronger their opposition to GMOs. Moreover, people with a higher degree of education generally show less bias in terms of gender perception of professionals or scientists.

Finally, the survey clearly demonstrates the influence of age on the perception of synthetic biology and related disciplines. The youngest and oldest respondents generally gave the lowest evaluations. The ones belonging to Generation X showed the most positive attitude towards these technologies.

This was the first large survey on the public's perception of synthetic biology. Given the sample size, it shows a significant difference in how these terms are perceived along with a robust difference in how women perceive female versus male scientists. These results would warrant further studies and analysis to explain these rather unexpected observations. Moreover, they can help to inform educational and outreach campaigns to address people's concerns over modern biotechnologies or to raise the interest in science, in particular among young women and girls.
